# ZnO Nano-Rod Devices for Intradermal Delivery and Immunization

**DOI:** 10.3390/nano7060147

**Published:** 2017-06-15

**Authors:** Tapas R. Nayak, Hao Wang, Aakansha Pant, Minrui Zheng, Hans Junginger, Wei Jiang Goh, Choon Keong Lee, Shui Zou, Sylvie Alonso, Bertrand Czarny, Gert Storm, Chorng Haur Sow, Chengkuo Lee, Giorgia Pastorin

**Affiliations:** 1 Department of Pharmacy, National University of Singapore, Singapore 117583, Singapore; taps2752@gmail.com (T.R.N.); pant.aakanksha@gmail.com (A.P.); hejunginger@yahoo.com (H.J.); wjgoh@u.nus.edu (W.J.G.); choonkeonglee@u.nus.edu (C.K.L.); zoushui@u.nus.edu (S.Z.); 2 Department of Electrical Engineering, National University of Singapore, Singapore 117583, Singapore; elewangh@nus.edu.sg; 3 Department of Physics, National University of Singapore, Singapore 117551, Singapore; elezmr@nus.edu.sg (M.Z.); physowch@nus.edu.sg (C.H.S.); 4 NUS Graduate School for Integrative Sciences and Engineering, Centre for Life Sciences (CeLS), Singapore 117456, Singapore; 5 Department of Microbiology, National University of Singapore, Singapore 117545, Singapore; micas@nus.edu.sg; 6 School of Materials Science and Engineering (MSE) & Lee Kong Chian School of medicine (LKCmedicine), Nanyang Technological University, Singapore 636921, Singapore; bczarny@ntu.edu.sg; 7 Department of Pharmaceutics, Utrecht Institute for Pharmaceutical Sciences, Faculty of Science, Utrecht University, Universiteitsweg 99, 3584 CG Utrecht, The Netherlands; G.Storm@uu.nl; 8 NUSNNI-NanoCore, National University of Singapore, T-Lab, Blk E3-05-29, 2 Engineering Drive 3, Singapore 117581, Singapore

**Keywords:** ZnO nano-rods, skin immunization, intradermal delivery

## Abstract

Intradermal delivery of antigens for vaccination is a very attractive approach since the skin provides a rich network of antigen presenting cells, which aid in stimulating an immune response. Numerous intradermal techniques have been developed to enhance penetration across the skin. However, these methods are invasive and/or affect the skin integrity. Hence, our group has devised zinc oxide (ZnO) nano-rods for non-destructive drug delivery. Chemical vapour deposition was used to fabricate aligned nano-rods on ZnO pre-coated silicon chips. The nano-rods’ length and diameter were found to depend on the temperature, time, quality of sputtered silicon chips, etc. Vertically aligned ZnO nano-rods with lengths of 30–35 µm and diameters of 200–300 nm were selected for in vitro human skin permeation studies using Franz cells with Albumin-fluorescein isothiocyanate (FITC) absorbed on the nano-rods. Fluorescence and confocal studies on the skin samples showed FITC penetration through the skin along the channels formed by the nano-rods. Bradford protein assay on the collected fluid samples indicated a significant quantity of Albumin-FITC in the first 12 h. Low antibody titres were observed with immunisation on Balb/c mice with ovalbumin (OVA) antigen coated on the nano-rod chips. Nonetheless, due to the reduced dimensions of the nano-rods, our device offers the additional advantage of excluding the simultaneous entrance of microbial pathogens. Taken together, these results showed that ZnO nano-rods hold the potential for a safe, non-invasive, and painless intradermal drug delivery.

## 1. Introduction

Efficient methods of drug delivery are of paramount importance in the successful treatment of medical conditions. Besides the traditional method of injection to deliver a certain drug molecule, there are a few non-invasive delivery routes (e.g., pulmonary, nasal, oral, transdermal), among which transdermal administration has the potential to determine a major impact on the way vaccines are administered. In fact, skin is highly immunogenic [1] by possessing a high concentration of dendritic cells which, when primed by allergens, migrate immediately to the lymph nodes initiating the required immune responses. Moreover, vaccines transported through the skin offer significant advantages, such as the possibility for a prolonged delivery and enhanced bioavailability for drugs with short half-life and, by avoiding injection, they provide increased patient compliance.

The main challenge associated with this administration route is represented by the skin’s outmost layer, the *Stratum Corneum* (SC), which is not sufficiently permeable to allow for effective transfer of medication into the bloodstream, and thus it often prevents the absorption of drugs [2]. Advanced technologies have introduced formulations that make use of chemical as well as physical or mechanical penetration enhancers (e.g., iontophoresis and electroporation) or even small needles of different shapes. Regarding this last aspect, microneedles have been proven to be of great interest recently, since they are able to create little holes across the SC and facilitate the penetration of drug and vaccine molecules [3]. Keeping the above information in mind, our goal was to carry out feasibility studies on the use of zinc oxide (ZnO) nano-rods grown successfully on silicon chips for the intradermal delivery of vaccines. In other words, we evaluated the ability of this unreported nano-rod based device, made up of nanometric rods, to act as an efficient tool for the penetration through the SC and the stimulation of the immunological response. Being a preliminary study, we utilized ovalbumin (OVA) as a vaccine prototype with the hope that the data obtained will be useful for future investigations of this novel device with regard to vaccines for pertussis, tetanus, influenza, etc.

## 2. Results

Among the realm of strategies aimed at protecting the body from external intruders by means of vaccination, the skin has evolved into a major immuno-competent organ, allowing the most effective immunization with the least amount of antigen [4]. In fact, even if their role has been recently regarded as controversial [5], the presence of Langerhans cells immediately beneath the *Stratum Corneum* (SC) seems to promote an immunoactive effect: once stimulated by allergens, these cells actively migrate to the lymph nodes initiating immune responses. However, this process is quite complex, since the SC is very effective at preventing not only the access of xenobiotics, infectious agents, and other substances into the body, but also the penetration of most drugs other than those with high potency (dose in milligrams or less), low molecular mass (1000 Da), and an optimal octanol-water partition coefficient. In our preliminary studies, we demonstrated that chips with ZnO nano-rods indeed facilitated the penetration of our vaccine prototype (Albumin) through the skin, while albumin only weakly stimulated an immune response, suggesting that the delivery of vaccine prototype from the chips requires further optimization. The detailed microscopy evaluation of human skin samples treated with such devices demonstrated that the vaccine molecule passed through the canals formed by the nano-rods.

**Figure 1:**
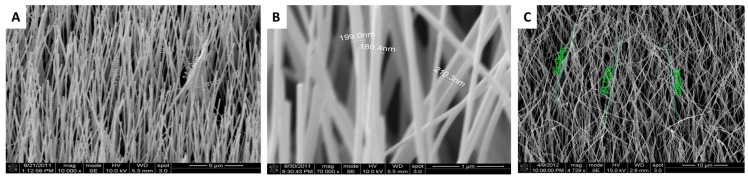
Scanning electron microscopy (SEM) images of the cross-section of nanochips. The two left panels (**A**,**B**) show vertically aligned nano-rods varying in length from 20 to 30 µm and width from ~100–200 nm. Rightmost panel (**C**) represents the nanochip with rods having a random alignment.

It was observed that uniform alignment of the nano-rods is one of the requirements to ensure that an intradermal delivery system works efficiently and with optimal penetration into the skin, i.e., it is able to pierce the *Stratum Corneum* and deliver a bioactive molecule through the transient pores formed by the rods without breaking. Therefore, our first effort was to obtain the optimal size and orientation of the nano-rods to achieve these goals. Batches of nano-rod based chips were produced through chemical vapour deposition (CVD) (Figure S1 in Supporting Information) [6,7], and imaged through scanning electron microscopy (SEM) (Figure 1). It was observed that the process of sputtering played a very important role in determining how the nano-rods grew. Indeed, most of fabricated nanochips had vertically aligned nano-rods (Figure 1A,B); however, some had rods showing a disordered pattern. The misalignment of the rods seen in Figure 1C is due to the underlying quality of the sputtered chips.

**Figure 2:**
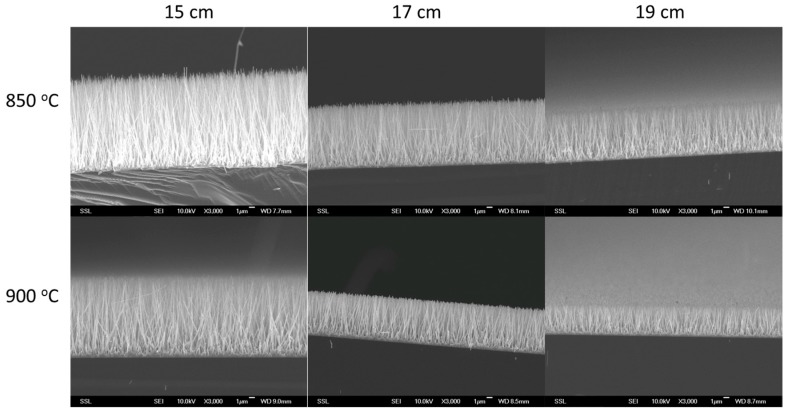
SEM images of vertically aligned zinc oxide (ZnO) nano-rods on silicon substrates using chemical vapour deposition (CVD), comparing the effect of temperature (850 vs. 900 °C) at distances of 15, 17, and 19 cm from the furnace source and powdered mix.

In order to have a uniform consistency in the alignment of the nano-rods, several runs of nano-chip fabrication were attempted and eventual variabilities were observed in the output through SEM. Using a CVD method, silicon wafers coated with a Zinc Oxide seed layer of approximately 200 nm were produced. The density, as determined by multiple SEM images (>10 photos at different positions) was about 6 nano-rods/μm^2^. The obtained chips were of about 1 cm × 1 cm dimensions and consisted of ZnO nano-rods with diameters of 80–300 nm and lengths within 40 μm, with nano-rods being the widest at the base. The distance between two nano-rods was about 0.3 μm. Not only sputtering played a crucial role, but other variables such as reaction time (2–6 h), temperature (850 vs. 900+ °C), distance of the chips from the powdered mix (10–20 cm), etc. had a relevant role in the fabrication of the rods (Figure 2). Optimization of the above parameters enabled the fabrication of a better batch of nano-chips, especially when reducing the reaction time from 6 h to 3 h in the tube furnace.

**Figure 3:**
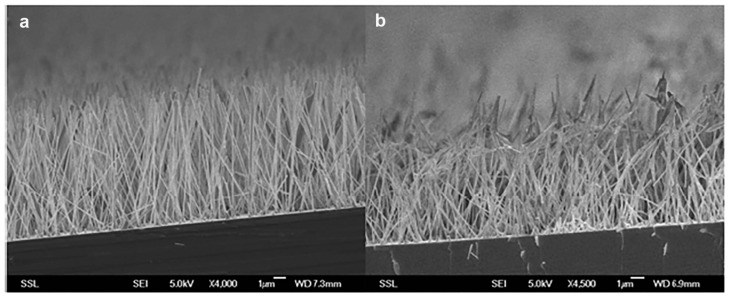
(**a**) 45° view. SEM image of ZnO nano-rods of about 20 μm aligned before the skin penetration studies; (**b**) 45° view. SEM image of ZnO nano-rods of 20 μm after the skin penetration. Vertical alignment is still visible, although the tips are slightly bent.

In particular, Figure 3 shows the SEM picture of the chip both before (on the left) and after (on the right) the skin penetration study. Although change in the shape of the nano-rods was evident from the pictures, it is important to note that the rods remained attached to the chip, since no reduction in their density was noticed along the sample. The change in the shape of the nano-rod tips is more likely due to the pressure exerted for the effective application of the chip on the skin sample. Future studies will be aimed at optimizing the release through the skin while avoiding excessive distortion of the nano-rods. Since the initial perforation of the skin, the subsequent penetration through the SC and the successful delivery of the vaccine ideally require vertically aligned nano-rods, and the chips having aligned nano-rods indeed attained these conditions.

With respect to the loading of the nanochips with vaccine prototypes, initial attempts at using two vaccine molecules, Vaxigrip and Infanrix, to optimally load the chips failed due to the problems of either the vaccine not being adsorbed properly to the rods or not desorbing well from the rods into the skin layer. Ovalbumin (OVA), a well-studied antigen for generating robust immune responses, was therefore adopted for subsequent experiments. Also, Bovine Serum Albumin (BSA) conjugated to a fluorophore (namely, fluorescein isothiocyanate, FITC) was used for animal experiments to track antigen migration and skin penetration studies.

The common ways of loading the antigen to the devices is either by dip coating or dry coating using a jet of gas to uniformly distribute the solution. However, the size of the nano-rods made these techniques unsuitable for our studies. Instead, the vaccine-containing solution was incorporated by dropping a specific volume on different areas of the chip and allowing the solution to air dry. After testing the nanochips, the estimation of delivery of a chip was done by washing the chips in Phosphate Buffer Saline (PBS) in a 24 well plate three times, allowing the OVA or BSA-FITC to desorb from the nanochips into the PBS. Finally, the protein estimation was done using the Pierce-BCA assay.

In addition, fluid samples were collected at different time frames through Franz-flow diffusion cells and subjected to the Bradford quantitative protein assay (OD recorded at 595 nm). They showed the presence of significant quantities of Albumin within the first 12 h, with a maximum peak already in the initial 3 h (Table S1, Supplementary Information). The quantity of protein decreased during the subsequent samples collected in the next 12 h, suggesting that the passage through the SC was almost complete within the first time slot. Regarding this last aspect, the SEM analysis of the samples after the penetration study indicated that the skin remained intact (image not shown) over the whole experimental process and thus confirmed the consistency of this observation. Moreover, a Bradford assay was employed in order to quantify the amount of Albumin effectively delivered through the skin, by the difference from the initial concentration and the amount collected after adsorption onto the chip. As reported in Table S2 (Supplementary Information), it was demonstrated that around 853 µg of Albumin-FITC were adsorbed onto the chip. Although the preparation of these initial chips was done manually, we observed that the value of absorbance at 529 nm (and, indirectly, the amount of protein that could be physically adsorbed on the chips) varied within a very small range between 0.0431 and 0.0479.

**Figure 4:**
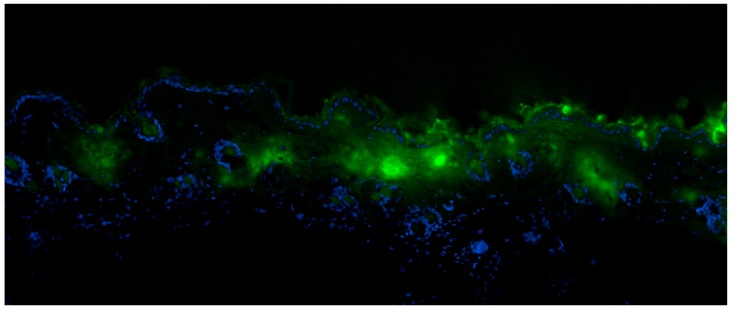
Sections of skin from histopathological studies that show Albumin-fluorescein isothiocyanate (FITC) penetration (green color) below the top layer (*Stratum Corneum*) of skin (stained with 4′,6-Diamidine-2′-phenylindole dihydrochloride (DAPI) in blue).

Skin penetration studies are required in order to measure the extent of absorption and penetration of the vaccine prototype across the SC of excised skin samples. In preliminary intradermal in vitro studies, we demonstrated that chips with Zinc Oxide nano-rods indeed facilitated the penetration of our vaccine prototype (Albumin) through the skin, while albumin stimulated an immune response, suggesting that the procedure can be easily extended for the delivery of a real vaccine. The detailed evaluation by microscopy of human skin samples treated with such devices demonstrated that the vaccine molecule was mainly absorbed through the canals formed by the nano-rods, whereas it was more stuck at the skin surface in between two consecutive needles (Figure 4). In support of this observation, no albumin was detected outside the area covered by the chip, thus confirming the necessity to have such a nanodevice to improve drug release.

The experiment on antigen migration to the lymph nodes was performed to assess whether the antigen was able to reach the viable epidermis below the SC by nano-rod application. Upon antigen exposure, the Langerhan cells of the skin immune system uptake the antigen and migrate to the nearest draining lymph nodes to present the processed antigen to the naive as well as effector T and B cells. Fluorescence-activated cell sorting (FACS) analysis revealed (Figure S1, Supporting Information) that the intensity of the antigen signal was maximal in the lymph nodes after 24 h of sub-cutaneous injection compared to the other time points, suggesting that it takes almost 24 h for the dendritic cells and other antigen presenting cells to capture the antigen and migrate to the lymph nodes where it is processed thereafter. Based on this positive control, an experiment was planned where the nanochips were loaded with 100 µg of BSA-FITC and applied to the shaven skin of the mice for 20 min with increasing pressure. Mice were injected sub-cutaneously with 50 µg of BSA-FITC.

**Figure 5:**
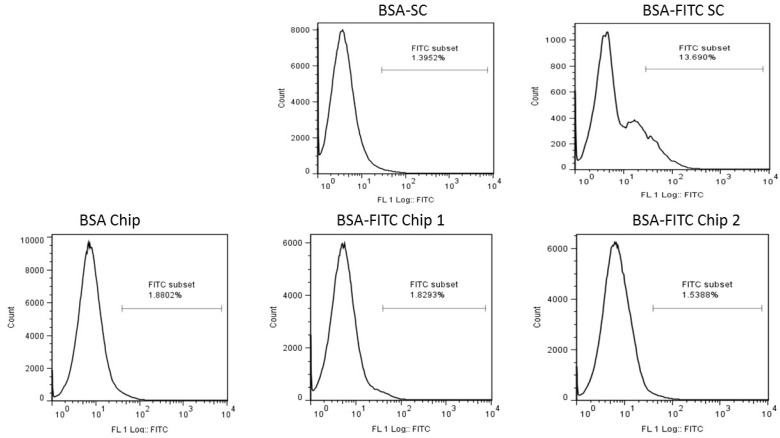
Antigen (BSA-FITC) migration to lymph nodes either subcutaneously or through the application of nano-rod devices (Chips 1 and 2). BSA alone, either injected subcutaneously (BSA-SC) or adsorbed on the nano-rod device (BSA Chip) acted as negative controls. After 24 h, BSA-FITC injected subcutaneously (BSA-FITC SC, positive control) showed dendritic cells’ migration to the lymph nodes, while BSA-FITC delivered by the two nano-rod devices (BSA-FITC Chip 1 and BSA-FITC Chip 2) showed negligible migration.

Result from the FACS analysis (Figure 5) indicated that the nanochip failed to deliver the antigen, in this case BSA-FITC, to the Langerhans cells in the skin below the SC. On estimation of the delivery of antigen, Chip 1 approximately delivered 10.6 µg of BSA-FITC and Chip 2 delivered 19.2 µg of the antigen. Compared to the dose of 50 µg delivered sub-cutaneously, the dosage given by the nanochips was insignificant.

In in vivo studies, the chips were treated with OVA. As indicated by Table S3 (Supporting Information), most of the protein (more than 70%) was released from the devices and diffused through the skin. It was important to note that, even though the chips were manually fabricated and cut, they showed a uniform appearance under SEM and a constant amount of protein adsorbed (narrow range of 450–503 μg of Albumin/chip). This last aspect represents a crucial point for future scale up of the devices.

Moreover, amidst the methods aimed at measuring skin appearance and its eventual modification [8], Transepidermal water loss (TEWL) is widely used since it is a quick and non-invasive biophysical technique. Such a measurement might be useful to determine skin damage, or in our specific case, to evaluate the nano-rod-mediated-enhancement of skin permeation. Prior to the treatment on skin samples, TEWL values were about 6.30 g·h^−1^·m^−2^ (SD ± 0.7) (data not shown). After the application of the chip onto the skin, the TEWL values increased immediately above 8.57 g·h^−1^·m^−2^, which is a close value to the reported cut-off of 13 g·h^−1^·m^−2^ for in vitro experiments on skin permeation. After 5 min of rest, the following TEWL measurement already provided a much lower value (≤7.00), which decreased rapidly in the following measurements. TEWL was increased by at least 2.3 g·h^−1^·m^−2^, as a demonstration of the efficacy in penetrating the skin. Therefore, the chips were able to disrupt the SC barrier to a higher extent, while inducing no irritation on the treated area. On the other side, the subsequent decrease of TEWL values to normal ranges suggested that the holes, once formed, rapidly closed, thus minimizing both skin irritation and any adverse effect.

Finally, we performed a small trial to investigate the ability of the nano-rod devices to evoke an immune reaction when applied on the skin of BALB/c mice. In our case, the specific IgG antibody titers were determined in the sera of immunized mice divided into three groups (naïve, PBS-SC, and nanochips, respectively) (Table S4, Supporting Information).

**Figure 6:**
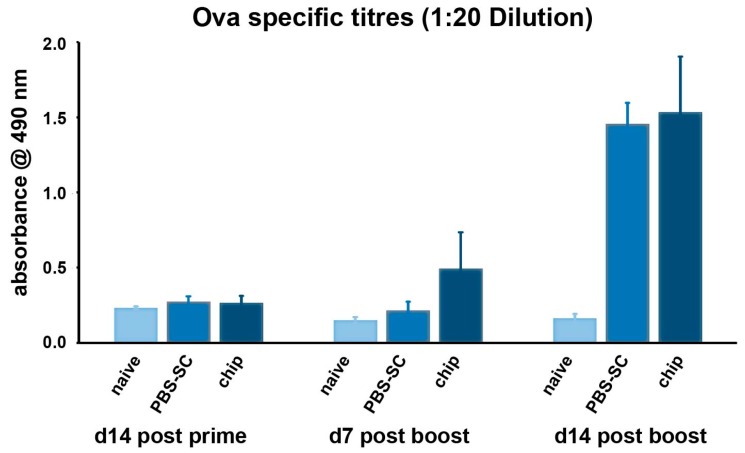
Ovalbumin (OVA)-specific antibody titres for mice immunized against OVA-Alum d14 after priming and d7 and d14 after booster immunization. The chip primed group had higher IgG titres at both day 7 and day 14 after the booster dose of OVA.

Anti-OVA IgG titres were comparable across all three groups on day 14 after immunization; that is, mice receiving OVA through the chips had similar titres compared to the PBS group and the naive mice group. On day 7 after the boost, the titres of the chip primed group were elevated compared to the naive and the PBS control group, and a similar trend was observed after day 14 of the boost, as sera IgG titres for the chip group were more than that for the PBS control group (Figure 6).

The amount of OVA necessary to induce proper immunization in mice is about 100 μg (our positive control). On the basis of previous results with the calculation of the antigen released from the chip (450–503 μg of Albumin/chip), we may assume that at least 1/5 of the whole dose has reached the desired target, but how much antigen remained entrapped in the SC and how much reached the epidermis with the Langerhans cells still have to be experimentally verified.

Although immunization studies demonstrated a weak antibody response, these initial data, together with the proposed ameliorations, suggest that further development of this work might result in an increased pharmacological effect of drugs delivered through the skin.

## 3. Discussion

Skin provides a highly complex and varied immune network comprised of various antigen presenting cells. Hence, the transdermal route of vaccination is a suitable alternative to conventional vaccination methods making use of syringe needles which, although effective, have proven to be phobia-inducing and highly disconcerting to a wide population [9]. Recent advances in the field of a more superficial, intradermal vaccination follow a study by an Australian research group that has devised a nanopatch consisting of arrays of densely packed projections with a defined geometry and distribution designed to physically target vaccines directly to thousands of epidermal and dermal antigen presenting cells (APCs). They have shown effective immunization against West Nile virus and Chikungunya virus through these nanopatches, further necessitating the need of extensive research for developing nanodevices for intradermal vaccination [10].

The purpose of our study was to fabricate nanodevices that reach the viable epidermis, which lies just below the layers of thick and hardened non-living SC. Our device was able to penetrate this layer (of about 20 μm in thickness) to reach the antigen presenting cells, namely Langerhans cells, in the viable epidermis.

We were successful in fabricating ZnO nano-rods of about 35–40 μm in length and 100–300 nm in diameter. The choice of zinc oxide was due to the lower costs of manufacturing the chips, biocompatibility, and safety of the substance to human cells. With respect to this last aspect, although ZnO nanoparticles have shown toxic and mutagenic effects on keratinocytes and fibroblasts [11,12,13,14], it is worth mentioning that our system is not designed to remain inside the skin. Indeed, if by accident the nano-rods broke into the *Stratum Corneum*, as this is a dead skin layer, no irritation would be expected to occur. Conversely, the broken parts would be easily extruded by the continuous regenerative process of the skin. By simply using finger tips to press the nanochips, it is possible to transfer these nano-rods into the viable epidermal layer without disrupting their shape.

Moreover, ZnO offers the advantage of being extremely versatile: the production by CVD is suitable for a variety of shapes and applications, depending on the need to use nano-sized fibers, rods, balls, or sheets. The reaction time proved to be critical in the growth of these rods. With several trials we were able to reduce the reaction time from 6 h to 3 h for aligned nano-rods. However, through confocal and immunization experiments, it was inferred that low levels of drugs/vaccines could be delivered by these nano-rods.

This was also confirmed by the lack of antigen migration to lymph nodes when delivery was performed through our nano-rod devices. There may be a few reasons why there was no antigen signal in the lymph nodes of the mice receiving priming with nanochips. First, the antigen delivered was very low as compared to the positive controls that received about 50 μg (while the nanochips could only deliver about 20 μg). Secondly, even from this dose of 20 μg, one should not expect that 100% of delivery occurred as some of the antigen may have been stuck at the SC layer and therefore did not completely reach the deeper layers of the viable epidermis. This is supported by the observation that most of the nano-rods deformed on applying pressure to the chips. Since not all the nano-rods in the chips are uniformly aligned, the penetration may not have been optimal. Third, the Langerhans cells populating the viable epidermis have different migratory properties compared to the dendritic cells found in other sites. Hence, more studies are warranted to assess the mechanism at the basis of such phenomenon.

Overall, the preliminary data derived from in vivo experiments and measuring the TEWL clearly showed that an increased water flux was obtained after application of the nanochip and that the viable epidermis was reached (even though not in an optimal way) with the short nano-rods of only 20 micrometers in length. The experiment also indicated that only 25 min after removal of the chip, the TEWL was reduced again to the normal values of the untreated skin, confirming that the created nanopores across the SC were immediately closed again; by this fact, a microorganism-mediated inflammation of the skin is minimized.

The resulting IgG titers from the in vivo experiments, which in our study were only enhanced by about 50%, could be attributed to the weak immunogenic properties of OVA [15]; as a proof of that, this vaccine prototype is usually associated to suitable adjuvants like chitosan and trimethyl chitosan (TMC) [16] in immunogenic studies. Moreover, it could be argued that most probably the dose reaching the viable epidermis was not sufficient to induce any significant response in the treated mice.

The OVA-alum immunization experiments give us a hint that the nanochips may work as a tool for delivering the antigen to the viable epidermis of the skin. The results provide some evidence that the nano-rods are priming the immune system. However, the overall levels are not significantly high, which may be due to the different quantities of OVA delivered by different nano-rods (Table S3). The level of priming was insignificant as compared to conventional vaccination means. One plausible explanation could be provided by the coating of the nano-rods, which was possibly insufficient to trigger the desired response in mice.

Nonetheless, the dimensions of our nano-rod devices are very attractive since most of the current reports in the intradermal vaccination area use microarray systems that have microneedles and these have not been sufficiently studied for the possibility of the spread of infections as they cause bigger perforations in the skin and also target the dermis. On the other hand, our nanodevices that only reach the viable epidermis leave the blood vessels present in the dermis unharmed and also have lower chances of infections following immunization.

On the basis of the precious role expected from these nanometric devices and our preliminary results, it is reasonably envisaged that these nano-rod systems will soon pave the way for an efficient intradermal delivery of vaccines.

## 4. Materials and Methods

### 4.1. Chemicals

Albumin-FITC (Albumin, Fluorescein isothiocyanate conjugate from bovine, A9771), goat anti-mouse IgG conjugate (A4416), and aluminum hydroxide gel (Alum, A8222) were bought from Sigma Aldrich (Singapore). Fetal bovine serum (FBS, research grade) was bought from HyClone (Cramlington, Northumberland, UK). Phosphate buffer saline (PBS, ultrapure grade) was bought from 1st BASE, Singapore. Tween 20 (polyethylene glycol sorbitan monolaurate, A R Grade) was bought from the laboratory supplies store at the National University of Singapore (NUS), Singapore. Endograde Ovalbumin (lyophilized) was bought from Hyglos GmbH, Bernried, Germany.

### 4.2. Fabrication of ZnO Nano-Rods

Before growing ZnO nano-rods on the silicon chips, the chips had to be treated with a process of sputtering which involves the coating of a thin film of ZnO onto the chips. The chips were sputtered and stored in a dry cabinet to prevent oxidation which may affect the growth of the nano-rods adversely. Nanochips with dimensions of 1 cm × 1 cm with ZnO solid rods growing with a vertical alignment to lengths of above 20 µm and diameters of 100–300 nm were fabricated using the chemical vapour deposition technique [6,7]. For this purpose, a ZnO and Graphite mixture was prepared in the ratio of 1:1 by grinding the mixture in a mortar and pestle for about one hour until it reached a uniform consistency. This powder mix was used for preparing the ZnO nano-rods. For making the nano-rod devices, 0.40 g of the ZnO:graphite powder was weighed and placed in a glass tube at the bottom. From the bottom of the tube, two silicon chips were placed at a distance of 15–17 cm and the tube was then kept in the tube furnace to grow the nano-rods by vapour deposition. The tube was placed in the machine and given a run of 6 h at 850 °C. Afterwards, the time was reduced to 3 h to obtain firmer and aligned nano-rods.

After the run, the chips were collected and analysed by SEM to verify their dimensions. The nano-rod chips were visualized through SEM (JEOL JSM-6701F field-emission scanning electron microscope) (NUS Chemistry Department, Singapore) and fluorescence microscopy (Nikon AZ-100) (Nikon Imaging Centre, Singapore), both before and after the in vitro skin permeation study to analyze the integrity of the nano-rods on the silica support and to determine the condition of albumin-FITC adsorbed onto the nano-rods. The human skin samples were visualized under a Nikon Eclipse Ti-E (Nikon Imaging Centre, Singapore) inverted microscope with a Nikon C1si laser scanning (Nikon Imaging Centre, Singapore) confocal for the depth and uniformity of the penetration.

### 4.3. In Vitro Intradermal Delivery of Vaccine Prototypes

Skin permeation studies were conducted to investigate the degree of absorption of Albumin-FITC by the skin samples from the Singapore General Hospital (SGH), with prior consent of the donor (human abdominal skin samples were obtained from a 22-year-old Indian lady from the SGH). In vitro permeation studies were carried out using amber glass Franz diffusion cells and the skin sample (2 cm by 2 cm) was analyzed under Fluorescent (Nikon AZ-100) and Nikon C1si Confocal Laser Scanning Microscopy. Before performing the in vitro studies, the SC was separated from the connective tissues of the human skin, to avoid interference in the experiments; this was achieved by immersing the whole skin in 60 °C water for 10 min, followed by careful removal of the SC [17]. Prior to experiments, these membranes with the top of the SC sides up will be floated over PBS (buffer). The chip, embedded with the vaccine, was applied for 5–10 min on the skin samples through a gentle fingertip pressure, in order to release the drug candidate. Franz flow-through type diffusion cells were used for the skin penetration study [17]: briefly, the chip was mounted onto the upper part with the nano-rods facing the collector chamber. SC layers were mounted onto the collector compartment facing the nano-rods. A solution of 500 mL of PBS was placed in the reservoir bottle and allowed to flow through the collector compartment at 0.50 mL/h. The collector solution was thoroughly degassed to prevent the formation of bubbles beneath the membrane. The temperature of the cells was maintained at 37 °C by a heater/circulator (Haake, Vreden, Germany). The PBS solution was pumped by a 16-channel peristaltic cassette pump (Ismatec, Wertheim, Germany) continuously through the collector compartment and drained into the test-tube sitting in the fraction collector (ISCO Retriever IV, Terre Haute, IN, USA). Liquid samples were collected at 4 h intervals for protein assay. Experiments were carried out in triplicate, and skin samples were analyzed under a Nikon Eclipse Ti-E inverted microscope. The quantification of the vaccine, both adsorbed onto the chip and delivered from the nano-rods, was determined through Bradford analyses, upon preparation of a standard curve. For the quantification of the vaccine, 20 µL of protein samples were added with Bradford reagent [18] and the OD was taken at 492 nm using a microplate reader (Spectra count, Perkin Elmer, Waltham, MA, USA). This OD was subsequently compared with the standard curve to obtain the appropriate protein concentration.

### 4.4. Antigen Migration to the Lymph Nodes

The antigen, BSA-FITC (bovine serum albumin conjugated with fluorophore FITC), was delivered through nanochip application on the skin of mice by measuring the FITC signal in the draining lymph nodes. The nanochips were manually inserted onto the shaved part of the mice’s skin with a gradual increase of pressure, in order to avoid premature bending of the nano-rods. As the application onto the skin was done manually, a control experiment (Figure S2) was done to assess the ability to efficiently and reproducibly pierce the skin and determine the time point at which the lymph nodes had to be harvested for FACS analysis. PBS was injected sub-cutaneously as a negative control and the positive control included mice injected sub-cutaneously with BSA-FITC with a dose of 50 μg.

### 4.5. In Vivo Skin Penetration Studies and Determination of Immune Responses

Female BALB/c mice, of 6–8 weeks of age at the beginning of the experiment, were bred in the animal facility (BRC Biopolis, Level 1, Singapore) with free access to rodent chow and water and were analyzed at 8–12 weeks of age. They were used in accordance with the guidelines of the National Research Council, Singapore (protocol number R14-608).

One week was chosen as a suitable interval of time to allow the mice to produce detectable amounts of antibodies, thus confirming the effectiveness of the nano-rod-based device on inducing an immune response.

These studies were performed on four groups of five mice, for a total of 20 mice.

Group I: mice not immunized (basal control);

Group II: mice subjected to chips adsorbed with the vaccine; 

Group III: mice subjected to either empty chips or chips embedded with the Alum adjuvant only (negative control);

Group IV: mice subcutaneously administered with 100 μg of OVA-Alum in solution form (positive control).

*Sample collection:* Blood samples (0.2 mL per animal) were collected from the cut tail tip on day 0 (before immunization) and 7 (at the end of study), and they were allowed to clot overnight and were then centrifuged at 8000 G for 5 min at room temperature. Serum was collected for each mouse and kept separately at −20 °C until assayed. Immune responses to the vaccine prototype were analyzed after 1 week by Enzyme-Linked Immunosorbent Assay (ELISA) in order to determine the levels of the specific serum immunoglobulin G (IgG) antibody as described elsewhere [18]. Briefly, after coating with the antigen, microtiter plates were blocked with 1% BSA. IgG titers were estimated by comparing the optical density (492 nm) of the sample and control wells (sera of non-immunized mice). Samples were considered positive when their optical density was twofold higher than that of the same dilution of the control. The corresponding titers were expressed as the geometric mean of reciprocal serum dilutions.

Groups of mice were immunized intradermally with 100 μg of OVA-Alum loaded nano-rods. In parallel, groups of mice were injected subcutaneously (SC) with PBS only (PBS-SC). The mice were bled at day 14 after a single immunization. At day 15, a booster dose of 50 μg of OVA-Alum was administered sub-cutaneously to all the groups of mice. The mice were bled again at day 7 and day 14 after the booster dose.

## Supplementary Materials

The following are available online at http://www.mdpi.com/2079-4991/7/6/147/s1.
