# Supplementary material for: ZnO Nano-Rod Devices for Intradermal Delivery and Immunization
Source: Nanomaterials (Basel). 2017 Jun 15;7(6):147. doi: 10.3390/nano7060147 (PMC5485794; doi:10.3390/nano7060147)
Supplement: Supplementary file 1 [file nanomaterials-07-00147-s001.pdf]

ZnO nano-rod devices for intradermal delivery and immunization

Tapas R. Nayak[1], Wang Hao [2], Aakansha Pant [1], Minrui Zheng [3], Hans Junginger[1], Wei Jiang Goh [1,4], Choon Keong Lee [1], Shui Zou [1], Sylvie Alonso [5], Bertrand Czarny [6], Gert Storm [7], Chorng Haur Sow [3], Chengkuo Lee [2*] and Giorgia Pastorin [1,3,8,*]

Supplementary Materials

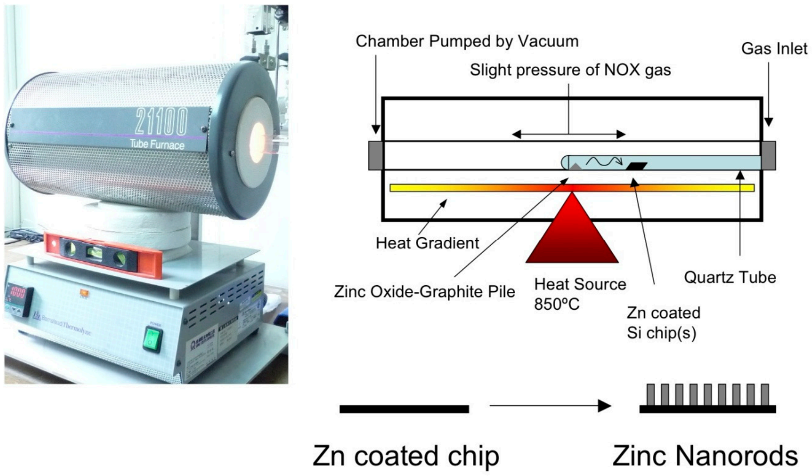

Figure S1. Left: example of a tube furnace for CVD. Right: Schematic representation of CVD process with zinc nanorods. Chip is inserted at a defined length from a pile of zinc oxide-graphite and the center is heated to 850°C. With vacuum, a slight pressure of NOX gas is allowed to reside in the chamber and the vaporized zinc oxide deposits on the chip to form nano-rods.

**Sample** (every 3 hours)	**Prot (µl)**	**Average OD at 595 nm ($x10^{-3}$)**	**Protein concentration (µg/ml)**	**Total quantity of Protein (µg)**
Blank	0	0.00	0.0	0.0
3 hrs	20	6.39	31.4	47.10
6 hrs	20	4.22	20.7	31.05
9 hrs	20	3.81	18.7	28.05
12 hrs	20	3.05	12.7	19.05
15 hrs	20	3.46	16.9	25.35
18 hrs	20	3.38	16.6	24.90
21 hrs	20	1.86	9.1	13.65
24 hrs	20	0.46	2.3	3.45
				Total: 192.60

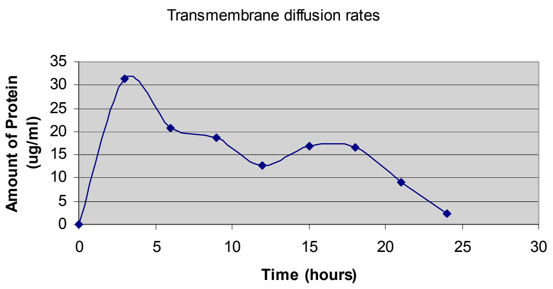

Table T1: Bradford quantitative albumin-FITC protein assay after skin penetration, calculated from the fluids collected every 3 hours.

Protein sample	OD (595nm)	Average OD	Protein quantity(mg)
Protein sample before adsorption on to chip (stock solution)	0.9486<br>0.9463<br>0.9545	0.9498 (SD 0.004)	5.397
Protein sample after adsorption onto chip	0.8012<br>0.7982<br>0.7967	0.7987 (SD 0.002)	4.544
Protein adsorbed onto the chip	-		0.853
Protein sample from washing of chip	0.0431<br>0.0461<br>0.0479	0.0457 (SD 0.002)	0.302
Protein sample collected from receptor chamber			0.193

Table T2: Analysis of quantity of albumin-FITC adosrbed on to the chip and amount released into the skin during the *in vitro* skin penetration study by Bradford assay. Where possible, the experiments were repeated in triplicates.

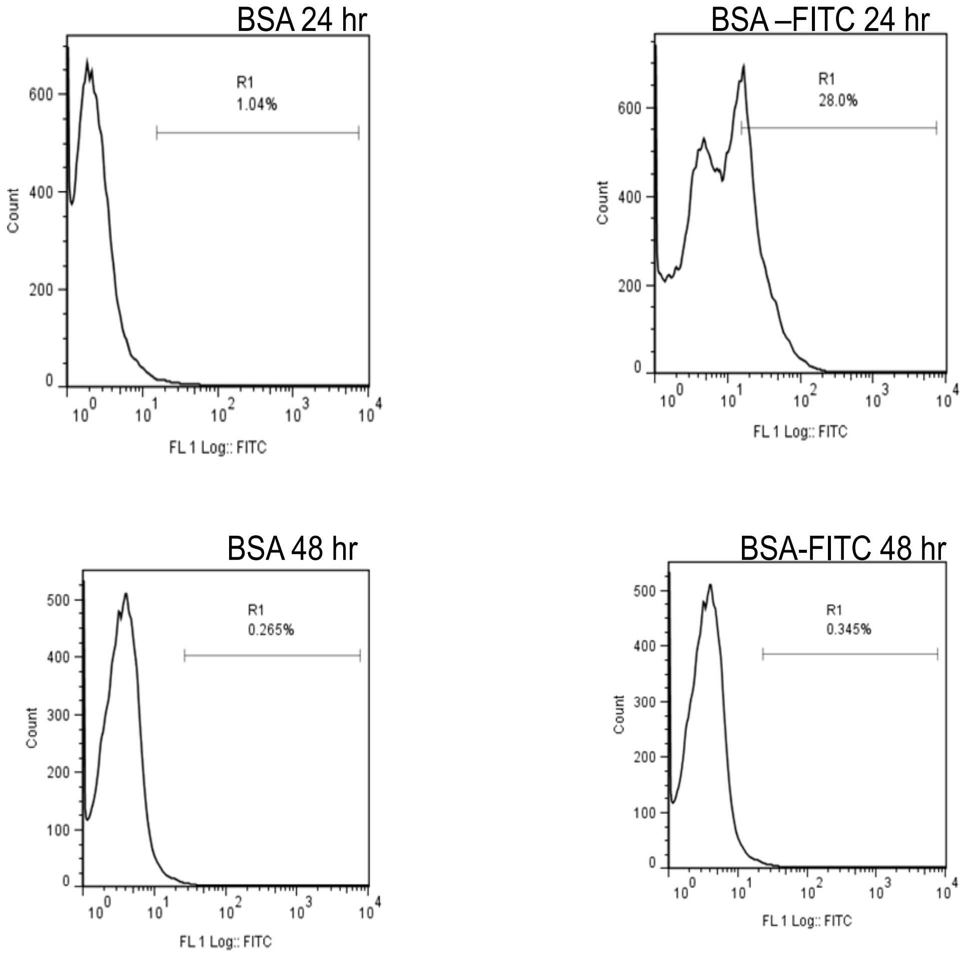

Figure S2: Antigen migration to the lymph nodes. After 24 hr the antigen BSA-FITC is delivered to the lymph nodes by dendritic cells in the immune system and this time point was taken as a reference while using the nanochips.

**Group**	**# mice**	**Treatment Day 0**	**Treatment Day 15**					
**Chip**	4	OVA-Alum Chip	OVA-Alum SC	**OVA-CHP1**	**OVA-CHP2**	**OVA-CHP3**	**OVA-CHP4**	**Average & SD of protein on chips 1-4**
**Naive**	4	-----	-----	**13.22**	**34.29**	**26.80**	**21.12**	**23.9±8.9**
**PBS-SC**	4	PBS	OVA-Alum SC					

Table T3. Estimation of the protein delivered by nanochips on day 0 of the treatment, as calculated by Pierce-BCA assay. Naïve: no treatment. SC: sub-cutaneous injection.

Antibody titres	naive1	naive2	naive3	naive4	AVE+SD naïve	PBS-SC1	PBS-SC2	PBS-SC3	PBS-SC4	AVE+SD PBS	chip1	chip2	chip3	chip4	AVE+SD chips
**d14post prime**	0.23	0.219	0.2405	0.24	0.232375 (SD 0.0101)	0.28	0.2685	0.229	0.317	0.2736 (SD 0.036)	0.266	0.2575	0.212	0.329	0.2661 (SD 0.0481)
**d7 post boost**	0.1365	0.1795	0.1445	0.146	0.1516 (SD 0.0190)	0.259	0.1265	0.255	0.2115	0.213 (SD 0.0615)	0.7595	0.1965	0.597	0.416	0.4923 (SD 0.2420)
**d14post boost**	0.148	0.2055	0.154	0.143	0.162625 (SD 0.0289)	1.4135	1.334	1.611	-	1.4528 (SD 0.1426)	1.7365	1.9975	1.1445	1.3535	1.558 (SD 0.3820)

Table T4. Total anti-OVA serum IgG titres at day 14 after intradermal priming with chips at day 7 and day 14 after sub-cutaneous booster immunization. Strangely, anti IgG antibody titres were not detectable for the PBS-SC4 sample after 14 days post-boost.
